# Adapting capillary gel electrophoresis as a sensitive, high-throughput method to accelerate characterization of nucleic acid metabolic enzymes

**DOI:** 10.1093/nar/gkv899

**Published:** 2015-09-13

**Authors:** Lucia Greenough, Kelly M. Schermerhorn, Laurie Mazzola, Joanna Bybee, Danielle Rivizzigno, Elizabeth Cantin, Barton E. Slatko, Andrew F. Gardner

**Affiliations:** From New England Biolabs, Inc., Ipswich, MA 01938, USA

## Abstract

Detailed biochemical characterization of nucleic acid enzymes is fundamental to understanding nucleic acid metabolism, genome replication and repair. We report the development of a rapid, high-throughput fluorescence capillary gel electrophoresis method as an alternative to traditional polyacrylamide gel electrophoresis to characterize nucleic acid metabolic enzymes. The principles of assay design described here can be applied to nearly any enzyme system that acts on a fluorescently labeled oligonucleotide substrate. Herein, we describe several assays using this core capillary gel electrophoresis methodology to accelerate study of nucleic acid enzymes. First, assays were designed to examine DNA polymerase activities including nucleotide incorporation kinetics, strand displacement synthesis and 3′-5′ exonuclease activity. Next, DNA repair activities of DNA ligase, flap endonuclease and RNase H2 were monitored. In addition, a multicolor assay that uses four different fluorescently labeled substrates in a single reaction was implemented to characterize GAN nuclease specificity. Finally, a dual-color fluorescence assay to monitor coupled enzyme reactions during Okazaki fragment maturation is described. These assays serve as a template to guide further technical development for enzyme characterization or nucleoside and non-nucleoside inhibitor screening in a high-throughput manner.

## INTRODUCTION

Nucleic acid metabolism, which includes DNA and RNA synthesis and degradation, is fundamental to all domains of life. Enzymes participating in DNA replication and repair have been widely studied for over a half-century (1). Standard methods to study nucleic acid enzymes generally measure synthesis or degradation of DNA or RNA by detection of radioactively or fluorescently labeled nucleic acid substrates. For example, a variety of simple methods have been developed to detect DNA polymerase activity and to screen for inhibitors. Fluorescent dyes that bind DNA have proven useful for DNA quantitation. An increase of PicoGreen or EvaGreen fluorescence upon dye binding to newly synthesized DNA can be used to measure DNA polymerase synthesis activity (2,3). Alternatively, DNA polymerase activity can be detected by fluorescence release of dye-quencher TaqMan probes or molecular beacons (4). Furthermore, DNA polymerase synthesis activity can be monitored by detection of pyrophosphate (PPi) or hydrogen ions that are released during synthesis (5,6). Raw fluorescence intensity changes of labeled oligonucleotides can be used to monitor activities of DNA polymerases, restriction endonucleases or DNA ligases (7,8). Both flap endonuclease (Fen1) and RNase H activity can be detected by changes in fluorescence polarization (FP) (9,10) or a coupled fluorescence resonance energy transfer (FRET) reporter system (11,12). These simple fluorescent techniques are useful for high throughput activity endpoint assays and to screen for enzyme inhibitors. However, reaction intermediates or side products are masked, therefore alternative assays which capture details of the reaction pathway are needed to gain a comprehensive understanding of nucleic acid enzyme activity.

Polyacrylamide gel electrophoresis (PAGE) is widely used to analyze both the size and distribution of substrates, intermediates and products. The structure and function of many nucleic acid enzymes have been characterized using these standard assays. Despite its utility, analysis by PAGE is relatively inefficient and limits the scope of enzyme analysis. The number of samples and conditions that can be analyzed on a single gel is restricted depending on gel percentage and substrate size. Furthermore, quantitation of products on PAGE gels is cumbersome and requires manual scanning and analysis. Therefore, analysis using PAGE gels constrains the number of reactions that can be performed and conditions that can be probed.

We describe an alternative method for nucleic acid enzyme characterization using high-throughput capillary gel electrophoresis. Capillary gel electrophoresis (CE) is a sensitive high-throughput, high-resolution system for nucleic acid analysis (13). In CE, fluorescently labeled nucleic acids are separated by size and charge, and detected by laser excitation. Sample loading and data acquisition is automated and rapid, allowing 96 samples to be analyzed in under an hour. Capillary gel electrophoresis first replaced slab gels in fluorescent Sanger DNA sequencing and accelerated high-throughput sequencing of the human genome (14,15). CE has also been used as an analytical tool for amplified fragment length polymorphism (AFLP) analysis, microsatellite analysis and single nucleotide polymorphism (SNP) detection (16). In this study, we multiplexed substrate design by both size and fluorescent color to simultaneously analyze multiple substrates, products and/or reaction intermediates in a single reaction, reducing analysis time and costs associated with enzyme characterization. In addition to the assays described here, we will also discuss the method's potential for enzyme discovery, characterization and engineering.

## MATERIALS AND METHODS

### Enzymes, oligonucleotides and reagents

All modifying enzymes, 9°N_m_ DNA polymerase, nucleotides and single-stranded M13mp18 DNA (ssM13) were from New England Biolabs (NEB, Ipswich, MA, USA). 9°N DNA polymerase D exonuclease minus (polD exo-) was purified as described previously (17). Oligonucleotide fluorescent dye absorbances, emissions, relative intensities and sequences are listed in Tables 1 and 2. Oligonucleotides were purchased from Integrated DNA Technologies (IDT, Coralville, IA, USA) or Life Technologies (Carlsbad, CA, USA). In general, primer-template substrates were prepared by annealing a fluorescently labeled primer and template (Table 2) in 1X ThermoPol buffer (20 mM Tris-HCl, 10 mM (NH_4_)_2_SO_4_, 10 mM KCl, 2 mM MgCl_2_, 0.1% Triton X-100, pH 8.8 @ 25°C) by heating to 95°C for 3 min followed by cooling to room temperature.

**Table 1.** Dye Absorbance maxima, emission maxima and relative intensities

Dye	Absorbance Max	Emission Max	Relative Intensity
FAM^TM^	494 nm	522 nm	100 RFU
VIC^®^	538 nm	554 nm	100 RFU
NED^TM^	546 nm	575 nm	40 RFU
TAM	560 nm	583 nm	25 RFU
PET^®^	558 nm	595 nm	25 RFU
LIZ^®^	638 nm	655 nm	50 RFU

Data from

‘DNA Fragment Analysis by Capillary Electrophoresis User Guide’ http://tools.lifetechnologies.com/content/sfs/manuals/4474504.pdf

**Table 2.** Oligonucleotide substrates

Oligonucleotide	Label	Sequence
FAM-Fen1 flap oligo (flap underlined)	3′-FAM	GTT AGT TCG AGC GTA ATG CCC TAT AGT GAG TCG TAT TAA GGT TGT AAA ACG ACG GCC AGT GCC AAG CTT GCA TGC CTG CA-**FAM**
Fen1 primer	none	CGC CAG GGT TTT CCC AGT CAC GAC G
FAM-extension primer	5′-FAM	**FAM-**CGC CAG GGT TTT CCC AGT CAC GAC
TAM-extension primer	5′-TAM	**TAM**-CGC CAG GGT TTT CCC AGT CAC GAC
FAM-ligation acceptor	5′-FAM	**FAM-**CGC CAG GGT TTT CCC AGT CAC GAC
Ligation donor	none	P-GTT GTA AAA CGA CGG CCA GTG CCA AGC TTG
Strand displacement template	none	GTC GAC CTG CAG GCA TGC AAG CTT GGC ACT GGC CGT CGT TTT ACA ACG TCG TGA CTG GGA AAA CCC TGG CG
Strand displacement DNA blocking oligo	none	GCC AAG CTT GCA TGC CTG CAG GTC GAC
FAM-incorporation template and exo primer	5′-FAM	**FAM**-AGT GAA TTC GAG CTC GGT ACC CGG GGA TCC TCT AGA GTC GAC CTG CAG GC
Incorporation and exo template	none	TTG CTC GTT TGC TGG GAG CCT GCA GGT CGA CTC TAG AGG ATC CCC GGG TAC CGA GCT CGA ATT CAC T
RNase H2 oligo	3′-FAM	rGrCrC rArArG rCrUrU rGCA TGC CTG CAG GTC GAC TCT AGA GGA TCC CCG GGT ACC GAG CTC GAA TT-**FAM**
Okazaki fragment blocking primer	3′-FAM	rGrCrC rArArG rCrUrU rGCA TGC CTG CAG GTC GAC TCT AGA GGA TCC CCG GGT ACC GAG CTC GAA TT-**FAM**

### Capillary gel electrophoresis

Capillary gel electrophoresis detects fluorescently labeled reaction products that are separated by size and charge as they migrate through a polymer filled capillary. CE samples are prepared by mixing 10 μl LIZ size standard diluted 1:40 in HiDi Formamide (Applied Biosystems) and 1 μl of fluorescently labeled reaction sample (between 0.15 nM and 20 nM) (18). The reaction sample and size standard fragments are injected electrokinetically into a 36 cm capillary array filled with Performance Optimized Polymer (POP7). High voltage electrophoresis (15 kV) ensures single base resolution. Typical run parameters are shown in Table 3. Migration of the fluorescently labeled sample and size standard fragments across the laser window is detected and recorded by a CCD camera on the instrument. A collection of DNA fragments labeled with a specific dye (LIZ) serves to create a standard curve that is used to calculate product size. For example, the 120 LIZ size standard includes 15, 20, 25, 35, 50, 62, 80, 110 and 120 nucleotide LIZ-labeled DNA fragments. In addition, it is recommended that fluorescently labeled synthetic substrate and product standards be used to verify their positions in CE assays. All reactions described below were separated by CE using a 3730xl Genetic Analyzer (Applied Biosystems) and fluorescent peaks were analyzed using Peak Scanner software version 1.0 (Applied Biosystems). The 3730xl Genetic Analyzer completes separation and analysis of 96 samples in less than 1 h.

**Table 3.** Typical run parameters for the 3730 xl Genetic Analyzer

Oven temperature	66°C
Buffer temperature	35°C
PreRun voltage	15 kv
PreRun time	180 s
Injection voltage	2.0 kv
Injection time	5 s
Run voltage	15 kv
Current stability	30 μA

### Data analysis

**Figure 1.**
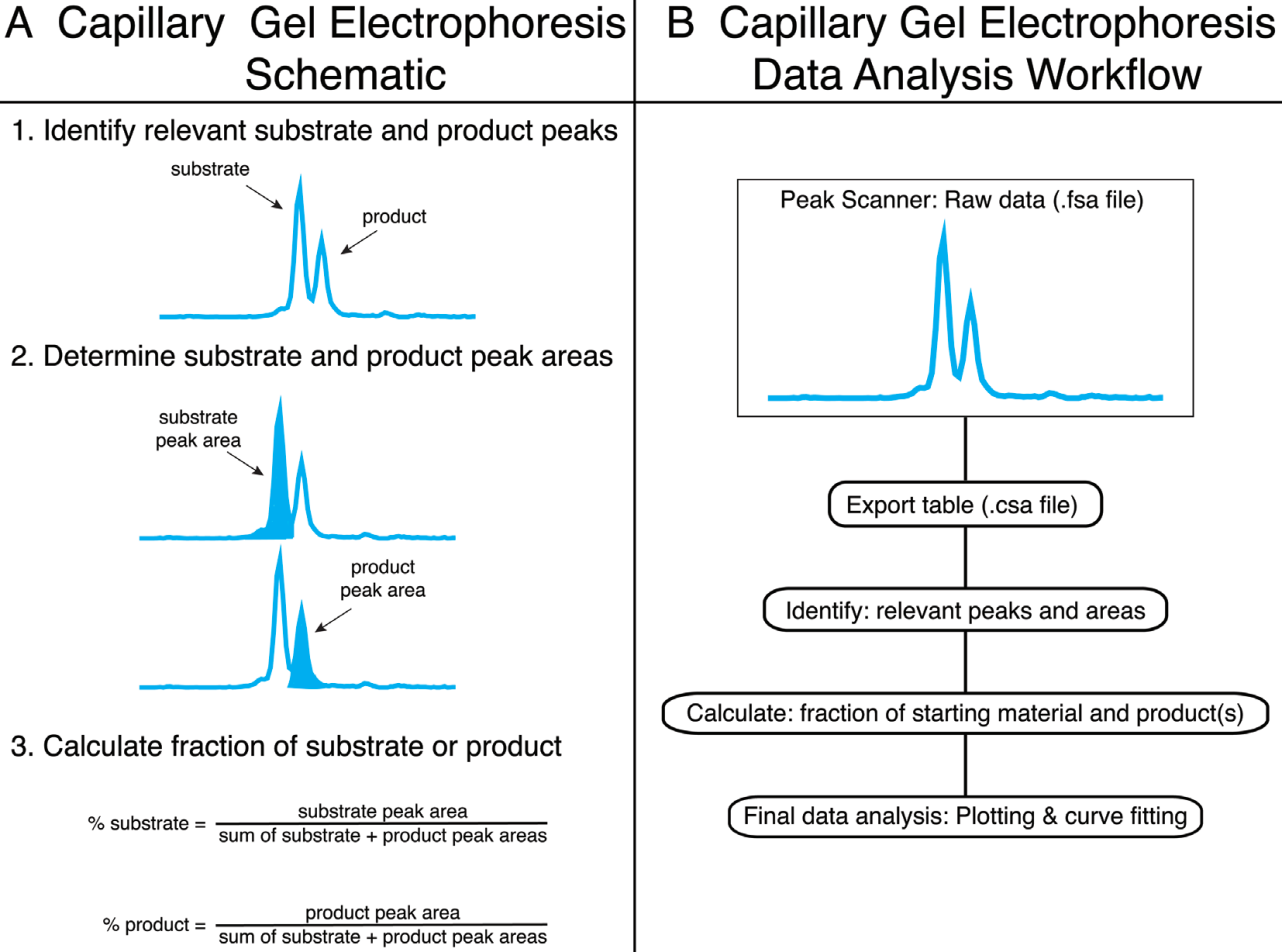
Capillary gel electrophoresis data analysis workflow. The CE data analysis workflow is illustrated schematically in panels (A) and (B). (**A**) Fluorescent reactions are resolved and detected by CE to identify substrate and product peaks. Next, peak areas are determined and fractions of substrate and products are calculated. (**B**) Peak Scanner software generates a raw CE data file (.fsa) that can be exported as a table (.csa file). Standard spreadsheet software can be used to extract peaks and areas, and to calculate fractions of substrates and products. Statistical graphic software is then used for final data analysis including plotting and curve fitting.

The CE data analysis workflow is outlined in Figure 1. Peak Scanner software (Applied Biosystems) displays CE peak fluorescence intensity and generates an .fsa file of raw data including peak dye, size, height, area, as well as information about peak start and end points. The .fsa file data can be exported as a comma-separated values (.csv) file and manually analyzed with spreadsheet software such as Microsoft Excel or Apple Numbers. Relevant data peaks are identified and peak areas are extracted (Figure 1). The fraction of substrate or products is calculated as described in Figure 1. If necessary, the product concentration can then be calculated by multiplying (starting substrate concentration)*(%product). Relative peak areas from each capillary should be analyzed independently since the amount of injected DNA varies between capillaries due to differences in loading efficiencies and buffer conditions. Manual analysis of substrates and products using Microsoft Excel or other spreadsheet software is straightforward, yet analysis of large data sets is time consuming (>1 h analysis per 96 reactions). To speed analysis, custom software tools or macros can be designed to rapidly extract and analyze large data sets (<10 s analysis per 96 reactions) and increase throughput. For final data analysis, data are plotted and curves fit using standard statistical or graphical software.

### General considerations for experimental design

The assays described below are examples designed to take advantage of the sensitivity, flexibility and automation of the capillary gel electrophoresis system to study nucleic acid enzymes. In this section, we discuss general experimental design parameters to maximize the potential of the system and any limitations that should be noted. The principles of assay design used here can be applied to nearly any enzyme system that acts on nucleic acids. Reaction substrates, intermediates and products can be labeled using different dye-labels having well separated excitation and emission spectra (FAM, TAM or NED, VIC and PET (Table 1)). Multiplexing oligonucleotide design by size and color allows simultaneous analysis of multiple substrates, products and/or reaction intermediates in a single reaction. The 3730xl Genetic Analyzer capillary gel electrophoresis instrument analyzes up to five dye-labels (FAM, TAM or NED, VIC, PET and LIZ as a size standard) in a single capillary using filter set G5. Fluorescent substrates can be detected between concentrations of <0.01 nM and 10 nM. Higher fluorescent substrate concentrations (>10 nM) saturate the CE detectors and cannot be reliably quantitated.

Dye-label fluorescence intensity may be sensitive to sequence context (7). In particular, nucleoside bases on the 5′ and 3′ side of the dye-labeled nucleoside may influence fluorescence intensity. Therefore, if possible, when designing substrates, dye-labels should be placed at a consistent sequence location to minimize fluorescence intensity differences. In addition, dye-labeled synthetic oligonucleotide controls having the same sequence as the analyzed substrates and products should be used as standards in each CE run. The standards verify both the expected elution times and relative fluorescent intensities of substrates, products and intermediates.

Nucleic acids are loaded electrokinetically onto the CE. During electrokinetic injection, salt anions compete with negatively charged DNA during loading. Therefore, as the salt concentration of a sample increases, less DNA will be injected into the capillary thereby decreasing the fluorescence signal. In addition, in the presence of formamide loading solution, excess salt can cause DNA to precipitate. Therefore, we recommend lowering the salt concentration before injection by either dilution with dH_2_O or size exclusion column chromatography. Salt concentrations between 0.25 mM NaCl and 2 mM NaCl caused no observable differences in typical fluorescence intensities or unusual suppression of individual product peaks.

There are several other important limitations of the CE method. Nucleotide composition and fluorescent dye structure affect the migration of the oligonucleotide fragment through the capillary. The migration of very short fragments (<10 nt) is dominated by the size of the dye label and therefore do not migrate according to their expected size (19). Because reactions are mixed with formamide prior to loading on the CE, DNA and any proteins will be denatured. Therefore, CE is not an appropriate method for electrophoretic mobility shift assays (EMSA).

### DNA polymerase activity assays

DNA polymerase pre-steady-state single nucleotide incorporation was monitored by extension of a fluorescently labeled primer/template (Table 2) as follows. PolD exo- (90 nM final concentration) bound to FAM-primer/template DNA substrate (30 nM final concentration) was mixed with dTTP (100 μM final concentration) in 1X Thermopol buffer using a Rapid Chemical Quench apparatus (RQF) (KinTek Corp., Snow Shoe, PA). After incubation at 60°C for 0.1–10 s, reactions were quenched with 50 mM EDTA. As a control, 5′-FAM template/primer was incubated with dTTP, without polD exo-, in the RQF. Reactions were analyzed by CE as described above. The product concentration was graphed as a function of time and the data were fitted to a single-exponential Equation (1) to obtain the rate of dTTP incorporation (*k*_obs_) using the nonlinear regression program Kaleidagraph (Synergy Software).

(1) \begin{equation*} [{\rm Product}] = {\rm A}[1 - \exp ( - k_{obs} {\rm t})]. \end{equation*}

DNA polymerase strand displacement activity was assayed by primer extension in the presence or absence of a downstream blocking primer. The strand displacement substrate was prepared as described above using oligonucleotides listed in Table 2. DNA polymerase (0.3 U/μl T4 or 0.8 U/μl Bst, Large fragment, final concentration), 15 nM of strand displacement substrate and 0.2 mM dNTP in 1X ThermoPol buffer were incubated for 30 min at 25°C or 50°C (for T4 and Bst DNA polymerases, respectively). Reactions were terminated by adding EDTA (100 mM final concentration). As a control, a 5′-FAM-primed template was incubated without polymerase as a 24 nt size standard. Reactions were analyzed by CE as described above.

The 3′-5′ exonuclease activity of 9°N_m_ DNA polymerase was tested by monitoring degradation of a 5′-FAM-labeled exo primer annealed to an unlabeled exo template (Table 2). Substrates were prepared as described above. Reactions were performed by adding 9°N_m_ DNA polymerase (1 Unit) to 10 μl of a solution containing 0.1 μM FAM-DNA substrate in 1X ThermoPol buffer. Samples were incubated at 72°C for 200 min and quenched with an equal volume of 0.5 M EDTA. Conversion of the fluorescently labeled DNA to shorter 3′-5′ exonuclease products was monitored by CE as described above.

### DNA ligase activity assay

DNA ligases seal nicked DNA during DNA replication and repair [reviewed in (20)]. To monitor DNA ligase activity, a DNA ligase substrate was annealed by mixing a 5′-FAM labeled acceptor oligonucleotide (50 nM) (Table 2), a 5′-phosphorylated unlabeled donor oligonucleotide (Table 2) (75 nM) and single-stranded M13mp18 (75 nM) (ssM13mp18) in 1X ThermoPol supplemented with 1 mM ATP as described above. 9°N DNA ligase (10 nM) was added and the reaction was incubated for 10 min at 60°C. Reactions were analyzed by CE as described above.

### Fen1 activity assay

Fen1 is a flap structure-specific endonuclease involved in DNA replication and repair [reviewed in (21)]. Fen1 activity was measured by assaying cleavage of a synthetic 3′-FAM labeled DNA flap substrate. Fen1 DNA substrate was prepared by annealing 3′-FAM labeled oligonucleotide (50 nM) containing a 40 nt flap, an unlabeled Fen1 primer (75 nM) (Table 2) and ssM13mp18 (75 nM) in 1X ThermoPol buffer as described above. The Fen1 flap DNA substrate (5 nM) was mixed with 9°N Fen1 (10 nM) in 1X ThermoPol buffer and incubated for 10 min at 60°C. Reactions were analyzed by CE as described above.

### RNase H2 activity assay

Ribonuclease H2 (RNase H2) is an endoribonuclease that preferentially nicks 5′ to a ribonucleotide in a DNA duplex. RNase H2 has been implicated in DNA repair and replication (22). RNase H2 activity was measured by monitoring cleavage of an RNA:DNA hybrid oligonucleotide (Table 2). The RNase H2 oligonucleotide (Table 2) was designed to mimic the structure of a downstream Okazaki fragment having a 10 nt 5′ RNA primer followed by 49 nt DNA and labeled with 3′-FAM. The RNase H2 substrate was annealed by mixing the 3′-FAM-RNase H2 oligonucleotide (50 nM) and circular ssM13mp18 DNA (75 nM) in 1X ThermoPol buffer as described above. The RNase H2 substrate (5 nM) was incubated with RNase H2 (10 nM) in 1X ThermoPol buffer for 10 min at 60°C. RNase H2 reactions were analyzed by CE as described above.

### GAN exonuclease activity assay

9°N GINS-associated nuclease (GAN) is a single-strand DNA (ssDNA) specific 5′-3′ exonuclease (23). GAN activity on single or double-stranded DNA with blunt, 3′-recessed ends or 3′-overhangs was monitored simultaneously using a four-color multiplexed fluorescence assay. Single-stranded DNA oligonucleotide (ssDNA) was labeled with an internal FAM. Double-stranded DNA was prepared by polymerase chain reaction (PCR). The forward PCR primer was fluorescently 5′-labeled for detection (either NED, PET or VIC). To create dsDNA having a 3′-overhang (5′-GCGC∧-3′), 5′-PET-labeled PCR product was digested with HaeII and purified. The 5′-VIC-labeled PCR product was digested with BstYI to generate a 3′-recessed end (5′-∧GATC-3′). To monitor 5′-3′ exonuclease activity on various substrates, NED-, PET-, VIC- and FAM-labeled substrates (10 nM) were incubated with 9°N GAN (100 nM) in 1X ThermoPol buffer for 15 min at 65°C. Reactions were analyzed by CE as described above.

### Dual label fluorescent Okazaki fragment maturation assay

During replication, Okazaki fragments are processed into contiguous lagging strands by the concerted action of a strand- displacing DNA polymerase, flap endonuclease and DNA ligase (24). A dual label fluorescence assay was designed to mimic *in vivo* Okazaki fragment maturation conditions and to observe different reaction intermediates simultaneously in a single tube. This assay detects two fluorescent dyes simultaneously and allows monitoring of DNA polymerase synthesis from a 5′-TAM-labeled extension primer (Table 2) and Fen1 plus DNA ligase processing of a 3′-FAM-labeled Okazaki fragment (Table 2). The Okazaki oligonucleotide mimics a downstream Okazaki fragment that has 10 nt of RNA at its 5′ end followed by 49 nt of DNA and a 3′-FAM label. There is a 20 nt single-stranded DNA gap between the extension and Okazaki fragment blocking primers. The Okazaki fragment maturation substrate was prepared by mixing the TAM-extension primer (50 nM), FAM-blocking primer (75 nM) and circular ssM13mp18 DNA (50 nM) in 1X ThermoPol buffer as described above. The FAM-labeled Okazaki fragment blocking primer is in molar excess over the template and extension primer so a fraction of the blocking primer remains unannealed and unreacted.

Typical Okazaki fragment maturation reactions were performed by mixing Okazaki fragment maturation substrate (5 nM), ATP (1 mM), dNTP (0.1 mM), 9°N proliferating cell nuclear antigen (PCNA) (200 nM), replication factor C (RFC) (400 nM), polB (10 nM), Fen1 (10 nM) and DNA ligase (10 nM) in 1X ThermoPol buffer. Reactions were incubated at 60°C for 2 min, terminated with EDTA (100 mM final concentration) and analyzed by CE as described above.

## RESULTS

Here we demonstrate the use of fluorescent oligonucleotide substrates and high-throughput CE to study enzymes acting on nucleic acids. CE facilitates analysis of up to an order of magnitude more reactions compared to traditional PAGE assays in a similar time frame. Typically, CE allows analysis of 96 reactions in similar or less time than analyzing 12 reactions on a typical PAGE gel system. In addition to increases in throughput, fluorescent substrates and products are quantitated automatically by CE software thereby reducing the steps needed to acquire results. We provide examples that illustrate how assays can be adapted for high-throughput CE analysis of a range of nucleic acid enzymes. The accompanying manuscript describes in detail how the high-throughput CE assay can be used to systematically and comprehensively characterize DNA ligase kinetics and fidelity (25).

### Capillary gel electrophoresis sensitivity and reproducibility

To determine the limit of CE detection, various concentrations of 3′-FAM labeled oligonucleotide were separated and detected by CE as described above. The linear range of detection was between 0.15 and 20 nM (Supplementary Figure S1). Reproducibility was evaluated by performing eight replicates of the Fen1 activity assay at six different Fen1 concentrations and analyzing variability (Supplementary Figure S2). Fen1 products generated were within 10% error for all replicates. Deviations from predicted data represent a mixture of effects, including incomplete or variable annealing of substrates, difficulties inherent in precise oligonucleotide concentration determination, potential context dependent variations in FAM fluorescence and potential CE injection differences. Thus we estimate a 10% error in our peak area determinations.

### Characterization of DNA polymerase activities

**Figure 2.**
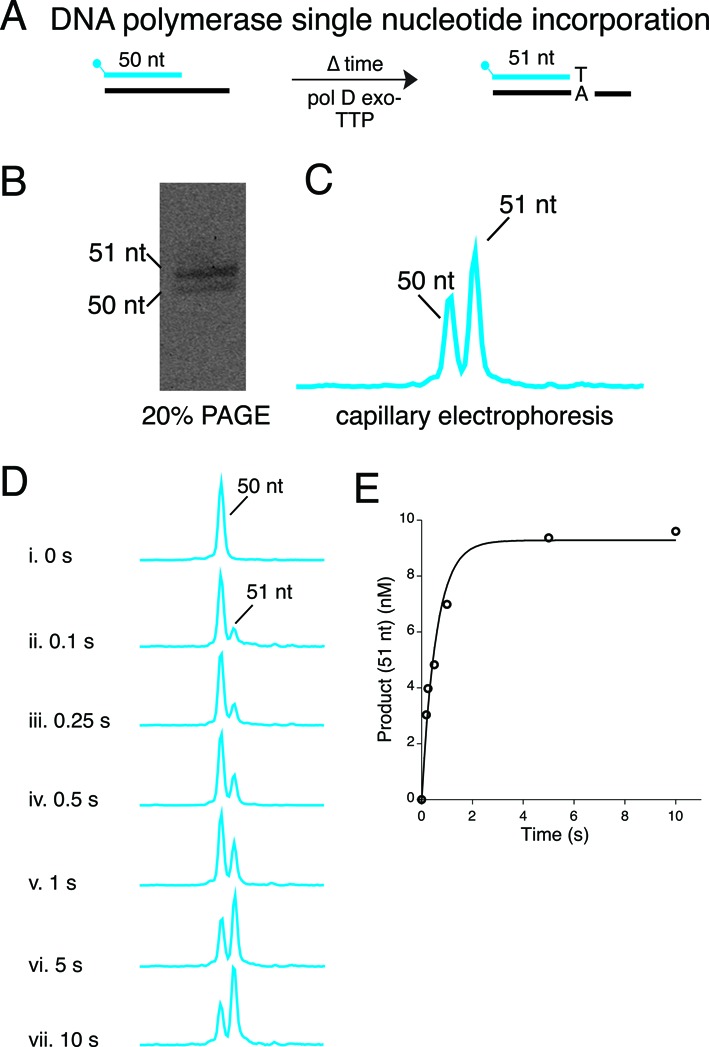
Characterization of DNA polymerase activities. (**A**) Single nucleotide incorporation was monitored by incubating 9°N polD exo-, a 5′-FAM-primer/template and TTP at 60°C followed by quenching with EDTA at various time points for 0–10 s. (**B**,**C**) Reactions were resolved by using 20% PAGE or capillary gel electrophoresis for migration comparison. (**D**) Each capillary gel electrophoresis time point was analyzed using Peak Scanner software (**E**) Reaction products from panel D were quantitated and plotted versus time (*k*_obs_ = 1.8 s^−1^).

**Figure 3.**
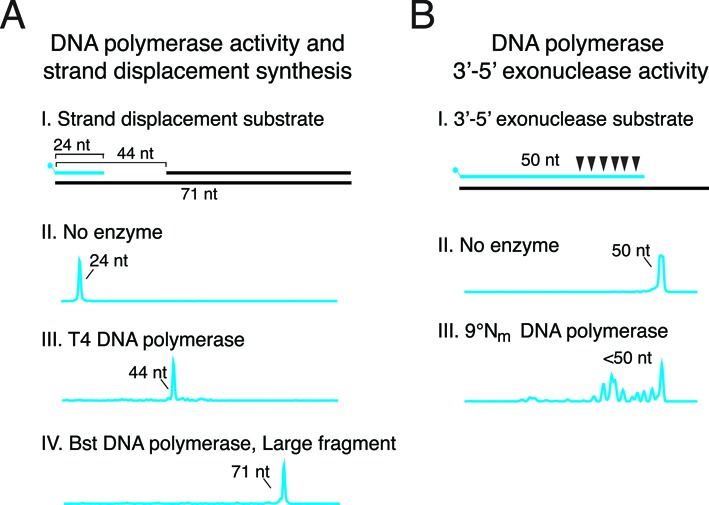
DNA polymerase strand displacement synthesis and 3′-5′ exonuclease activity. (**A**) T4 DNA polymerase or Bst DNA polymerase, Large Fragment strand displacement synthesis was monitored by extension of a 5′-FAM-labeled primer/template in the presence of a blocking primer 20 nt away from the extension primer. Reaction products were analyzed by capillary gel electrophoresis. (A III) T4 DNA polymerase synthesis halts upon encountering a downstream blocking oligonucleotide yielding a 44 nt extension product. (A IV) Bst DNA polymerase, Large Fragment strand displaces the blocking oligonucleotide to completely copy the 71 nt template strand. (**B**) 3′-5′ exonuclease activity (denoted by triangles) was monitored by incubating 9°Nm DNA polymerase and 5′-FAM-labeled primer/template. (B III) 9°Nm DNA polymerase 3′-5′ exonuclease activity degrades the 50 nt 5′-FAM-labeled primer resulting in shorter products (<50 nt).

DNA polymerases replicate DNA with extraordinary accuracy and efficiency (26). Substantial research has been devoted to understanding the structure and function of DNA polymerases. DNA polymerase kinetic studies have described the reaction pathway for correct nucleotide addition and mechanisms for discriminating against incorporation of incorrect nucleotides [reviewed in (27)]. Previous DNA polymerase kinetic studies used ^32^P-labeled primer/templates and PAGE to separate and detect polymerase synthesis products (28). As a high-throughput alternative to PAGE, we adapted CE to observe extension of fluorescently-labeled primer/templates. As discussed below, CE assays can be designed to monitor single-turnover kinetics (Figure 2), strand displacement synthesis (Figure 3) and 3′-5′ exonuclease activity (Figure 3).

Figure 2 illustrates using CE to analyze single-turnover DNA polymerase reaction kinetics as described in the Materials and Methods section. Because most single turnover rates for correct nucleotides are rapid (10 s^−1^ to 100 s^−1^), a rapid chemical quench instrument (RQF) is required for the collection of fast time points (>2.0 ms) (29). In this example, incorporation of dTTP (100 μM) by polD exo- was measured using an RQF to analyze reactions between 0 and 10 s. The 50 nt substrate and 51 nt dTTP extension product were resolved by PAGE or CE for comparison (Figure 2B). The concentration of product was quantitated and plotted versus time to derive the dTTP incorporation rate (*k*_obs_ = 1.8 s^−1^) (Figure 2E). The incorporation rate (*k*_obs_) was consistent with reported values in the literature (30).

To study strand displacement synthesis by a DNA polymerase, the extension of a 5′-FAM-primer was monitored in the presence of a downstream blocking oligonucleotide (Figure 3A). During polymerization, the presence of a downstream strand may block further polymerization or may be displaced by DNA polymerase strand displacement synthesis. ϕ29 DNA polymerase couples efficient DNA synthesis with strand displacement to synthesize very long DNA molecules (31). T4 and T7 DNA polymerases lack strand displacement activity and halt synthesis upon encountering a downstream strand (32). Previous studies used PAGE to monitor strand displacement synthesis on a gapped substrate (32–34). Instead, we adapted the strand displacement assay to allow analysis by CE using fluorescently-labeled extension primers. As expected, T4 DNA polymerase lacks strand displacement activity and stalls upon encountering the downstream blocking oligonucleotide leaving a 44 nt product (Figure 3A). In contrast, Bst large fragment DNA polymerase has robust strand displacement synthesis that displaces the downstream blocking oligonucleotide, completely copying the template strand (71 nt) (Figure 3A). The CE strand displacement assay is a simple, qualitative assay to determine if a DNA polymerase supports strand displacement synthesis. However, the DNA size detected by the CE is limited thus long strand displacement events (>800 nt) are outside the CE detection limit, therefore, other analysis methods such as PAGE should be used.

A subset of DNA polymerases also possess proofreading activity. If an incorrect nucleotide is incorporated, the 3′-5′ exonuclease activity of the polymerase removes the wrong nucleotide, giving the polymerase another opportunity to incorporate the correct nucleotide (35). In the absence of dNTPs, DNA polymerase 3′-5′ exonuclease activity removes dNMPs from the 3′ terminus regardless of the presence of a correct base or base mismatch. The 3′-5′ exonuclease activity of 9°N_m_ DNA polymerase was studied using a 5′-FAM-labeled primer/template without dNTPs. We observed exonuclease cleavage and therefore shortening of the 5′-FAM primer (Figure 2E). CE clearly resolves the formation of many exonuclease cleavage products, each of which can be quantitated. The pattern of 3′-5′ exonuclease degradation of a 5′-FAM primer in this study was consistent with previous data on 9°N_m_ 3′-5′ exonuclease degradation of a 5′-^32^P primer resolved by PAGE (36).

### Characterization of DNA ligase, Fen1 and RNaseH2 activities

**Figure 4.**
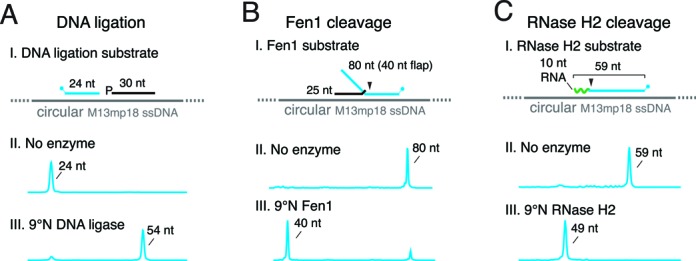
Characterization of DNA replication and repair enzymes by capillary gel electrophoresis. Replication enzymes DNA ligase, Fen1 endonuclease and RNase H2 were assayed for activity as described in Materials and Methods. All reaction products were separated and analyzed by capillary gel electrophoresis. (**A**) DNA ligation substrate was prepared by annealing a 5′-FAM-primer (blue), an unlabeled oligonucleotide with a 5′ phosphate (black) and a ssM13mp18 template. (A III) 9°N DNA ligase seals the 5′-FAM-primer and unlabeled oligonucleotide yielding a 54 nt product. (**B**) Fen1 substrate was prepared by annealing a 3′-FAMoligonucleotide (blue) having an unpaired 40 nt flap, an unlabeled oligonucleotide (black) and a ssM13mp18 template. (B III) Fen1 cleaves at flap structures generating a 40 nt product. (**C**) A 59 nt oligonucleotide having 10 nt of RNA on the 5′ end (green) and a 3′-FAM label was annealed to a ssM13mp18 template. (C III) 9°N RNase H2 cleaves the 10 nt RNA portion of the substrate leaving a 49 nt 3′-FAM-labeled product.

DNA ligase, Fen1 and RNase H2 play key roles during DNA replication, repair and RNA metabolism (24). DNA ligase seals DNA nicks created during DNA repair and Okazaki fragment maturation (37). We observe DNA ligation by CE through the joining of a fluorescent oligonucleotide donor (24 nt) and 30 nt unlabeled oligonucleotide acceptor, creating a 54 nt ligation product (Figure 4A). The accompanying manuscript is an in depth example of using the CE method to comprehensively characterize DNA ligase kinetics and fidelity. Fen1 is responsible for removing 5′-flaps that form during DNA repair and DNA replication. Using CE analysis, we observe the cleavage of an unpaired 5′-flap by Fen1 from a 3′-FAM-labeled flap substrate resulting in a 40 nt product (Figure 4B). The CE assay of Fen1 activity provides more detail into Fen1 cleavage sites and intermediates compared to end point FRET or FP techniques used previously (9,10) therefore accelerating enzyme characterization. Finally, we observe the activity of RNase H2 by the loss of the 10 nt RNA portion of a 3′-FAM-RNA/DNA oligonucleotide primer/template resulting in a 49 nt product (Figure 4C). Importantly, CE can be used to analyze both RNA and DNA substrates and products.

**Figure 5.**
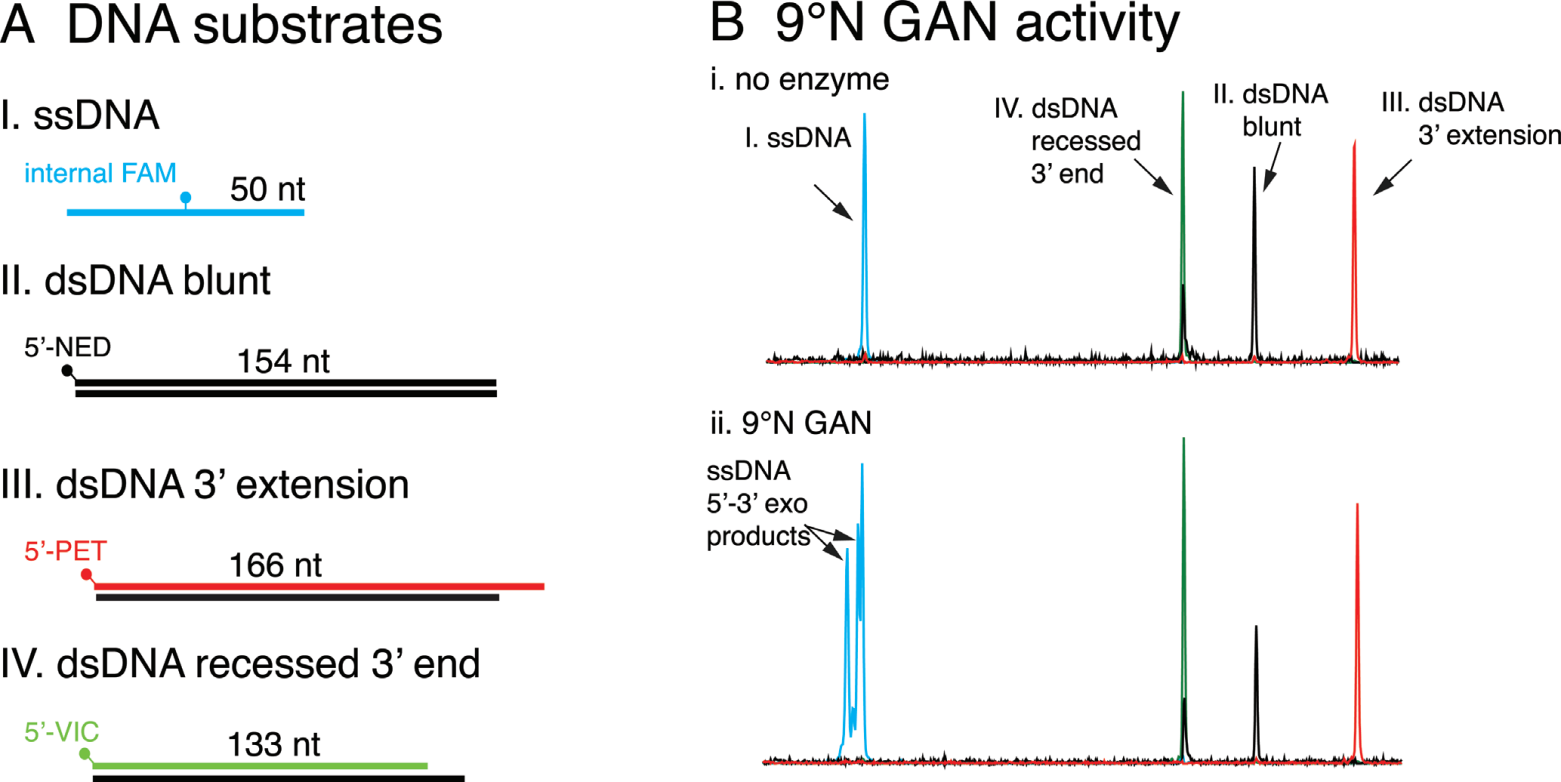
Characterization of GAN ssDNA 5′-3′ exonuclease activity by a four-color fluorescence assay and capillary gel electrophoresis. (**A**) To determine the GAN exonuclease substrate specificity, four fluorescently labeled substrates were prepared as described in Materials and Methods. (A I) ssDNA was internally labeled with FAM (50 nt; blue). (A II) Blunt ended dsDNA with a 5′-NED label (154 nt; black). (A III) dsDNA having a 3′-extenion with a 5′-PET label (166 nt; red). (A IV) dsDNA with a 3′-recessed end and a 5′-VIC label. (**B**) The four substrates are resolved by capillary gel electrophoresis as separate peaks having different fluorescence spectra. (B) Consistent with previously reported activity (23), 9°N GAN 5′-3′ exonuclease is active on ssDNA substrates as shown by FAM-labeled ssDNA 5′-3′ exonuclease products.

### Characterization of 9°N GAN exonuclease substrate specificity using a four-color fluorescence assay

GAN nuclease is a processive, single-strand DNA-specific exonuclease that degrades DNA in the 5′-3′ direction. GAN associates with GINS and a Family D DNA polymerase as part of the replisome, although its role during replication is not well understood (23). To characterize GAN substrate specificity and activity, we designed a multiplexed four-color fluorescence assay with a panel of ssDNA and dsDNA substrates. By combining four substrates into a single reaction tube, enzyme activity on all substrates can be analyzed simultaneously by CE. GAN only degrades ssDNA as detected by degradation of FAM-labeled ssDNA. GAN has no detectable activity on blunt, 3′-recessed or 3′-extension dsDNA (Figure 4). Due to the simultaneous detection of four different fluorescent labels by CE, substrates and the resulting products can be clearly identified. The GAN cleavage specificity observed here required a single CE capillary for analysis and is consistent with a previous study that used multiples lanes of a polyacrylamide gel (23).

### Characterization of Okazaki fragment maturation using a dual-label fluorescence assay

**Figure 6.**
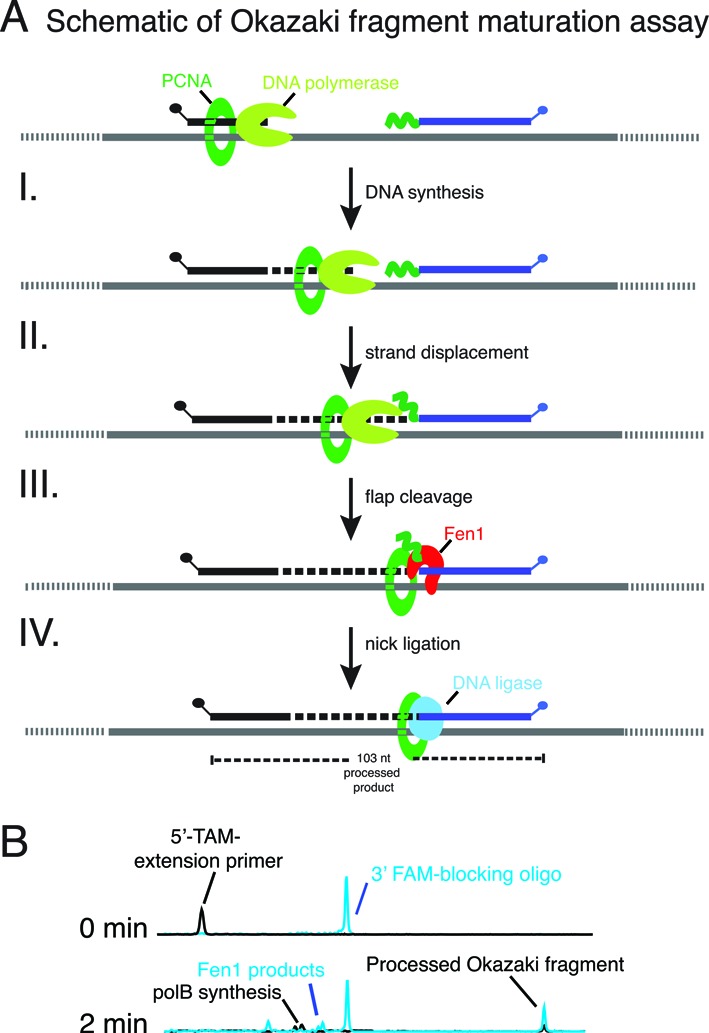
Characterization of Okazaki fragment maturation and detection of reaction intermediates by capillary gel electrophoresis. (**A**) A simplified schematic of Okazaki fragment maturation and expected results of capillary gel electrophoresis. (I) Together with PCNA/RFC, a DNA polymerase initiates synthesis from the 24 nt 5′-TAM-extension primer resulting in products longer than 24 nt. (II) DNA polymerase strand displacement of the downstream Okazaki fragment (blocking primer) creates flap structures that (III) are cleaved by Fen1 resulting in removal of the RNA portion. (IV) The remaining DNA:DNA nick is then sealed by DNA ligase to generate a dual 5′-TAM- and 3′-FAM-labeled processed Okazaki fragment (103 nt). (**B**) 9°N polB, Fen1, DNA ligase, PCNA and RFC were incubated with the Okazaki fragment maturation substrate at 60°C as described in Materials & Methods. Reaction aliquots were sampled at 0 and 2 min, quenched with EDTA and analyzed by capillary gel electrophoresis.

During lagging strand synthesis, short Okazaki fragments are synthesized discontinuously and are processed into an uninterrupted lagging strand. The process of joining Okazaki fragments includes the removal of ribonucleotides at the 5′ end of each Okazaki fragment, gap filling and ligating the resultant DNA nicks. Here we designed a dual-color fluorescence assay to monitor Okazaki fragment maturation (outlined in Figure 6). During this process, polB extends the 5′-TAM-labeled extension primer and strand displacement synthesis creates flap structures in the 3′-FAM labeled Okazaki fragment. Fen1 cleaves flap structures to generate a nick. Fen1 products are visualized as shorter 3′-FAM-labeled products (Figure 6). Finally, DNA ligase seals together the 5′-TAM-primer and 3′-FAM-labeled oligonucleotides to produce a processed 103 nt Okazaki fragment (Figure 6). Due to the dual-labeling assay design, both 5′-TAM- and 3′-FAM-labeled reaction substrates, intermediates, and products can be quantitated allowing a detailed understanding of Okazaki fragment maturation pathway kinetics.

## DISCUSSION

Analysis of fluorescent substrates by capillary gel electrophoresis offers a high-throughput and highly sensitive method to probe enzyme activities leading to a more comprehensive understanding of nucleic acid metabolism. Each of the examples presented here demonstrates the utility of CE for in-depth analysis of a variety of enzymes that act on nucleic acids. Using CE as an analytical tool fundamentally changes the scale and complexity of experimental design. Instead of performing a handful of reactions with a single substrate, many substrates can be monitored in the same tube simultaneously by varying substrate size and fluorescent labels. In addition, 96 reactions can be easily processed and analyzed in parallel, thereby generating a more comprehensive view of enzyme activity.

The CE assays can also be adapted to study virtually any enzyme that acts on fluorescent DNA or RNA oligonucleotides. In addition to the assays described here, fluorescent oligonucleotides can be designed to study DNA repair, recombination, restriction and modification and RNA metabolism. Fluorescent substrates containing DNA or RNA lesions or mismatches can be used to probe DNA repair activities of known DNA repair and replication enzymes such as DNA glycosylases including UDG, Fpg, AAG, OGG1, and SMUG1 or endonucleases III, IV, V, VIII, and APE1. The same panel of substrates can also be used to screen cell extracts for novel repair enzyme activities in base excision repair, DNA mismatch repair or nucleotide excision repair. Similarly, mechanisms of homologous recombination and non-homologous end joining can be studied by monitoring recombination of labeled substrates. Restriction endonuclease site specificities and reaction parameters can be determined using a panel of fluorescently labeled substrates containing variable recognition sequences. Analysis by CE is not limited to DNA and is also an excellent method to study RNA metabolic enzymes. For example, reverse transcriptase extension of fluorescent primers on RNA templates can be monitored as well as RNA degradation by RNase H.

Because of high-throughput experimental design, CE assays can also be adapted to screen therapeutic inhibitors of DNA replication enzymes associated with cancer, autoimmune conditions and viral or bacterial infection (38). For example, clinically relevant drug targets such as herpes simplex virus (HSV) DNA polymerases can be screened for inhibition by nucleotide inhibitors using the same assay design principles described here.

## Supplementary Material

SUPPLEMENTARY DATA
