# Supplementary material for: Adapting capillary gel electrophoresis as a sensitive, high-throughput method to accelerate characterization of nucleic acid metabolic enzymes
Source: Nucleic Acids Res. 2015 Sep 13;44(2):e15. doi: 10.1093/nar/gkv899 (PMC4737176; doi:10.1093/nar/gkv899)
Supplement: SUPPLEMENTARY DATA [file supp_44_2_e15__index.html]

Adapting capillary gel electrophoresis as a sensitive, high-throughput method to accelerate characterization of nucleic acid metabolic enzymes — Adapting capillary gel electrophoresis as a sensitive, high-throughput method to accelerate characterization of nucleic acid metabolic enzymes — SUPPLEMENTARY DATA 

# Adapting capillary gel electrophoresis as a sensitive, high-throughput method to accelerate characterization of nucleic acid metabolic enzymes

## SUPPLEMENTARY DATA

- SUPPLEMENTARY DATA
