# Supplementary material for: Adapting capillary gel electrophoresis as a sensitive, high-throughput method to accelerate characterization of nucleic acid metabolic enzymes
Source: Nucleic Acids Res. 2015 Sep 13;44(2):e15. doi: 10.1093/nar/gkv899 (PMC4737176; doi:10.1093/nar/gkv899)
Supplement: SUPPLEMENTARY DATA [file supp_gkv899_nar-01317-met-k-2015-File008.pdf]

Supplemental Figures
Greenough, et al.
"Adapting capillary gel electrophoresis as a sensitive, high-throughput method to accelerate characterization of nucleic acid metabolic enzymes"

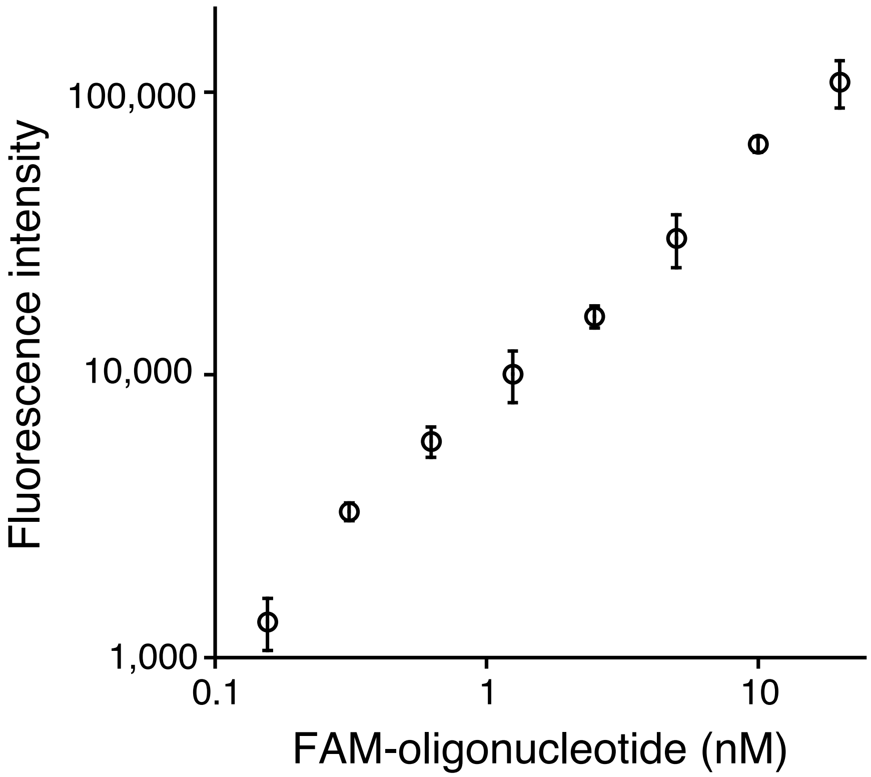

Supplemental Figure 1. FAM-oligonucleotide dynamic range of detection. A FAM-labeled single stranded oligonucleotide (FAM Fen1 flap oligo) was serially diluted in water (20 nM to 0.01 nM). The linear range of detection (between 0.15 nM and 20 nM) is plotted above as a log plot.

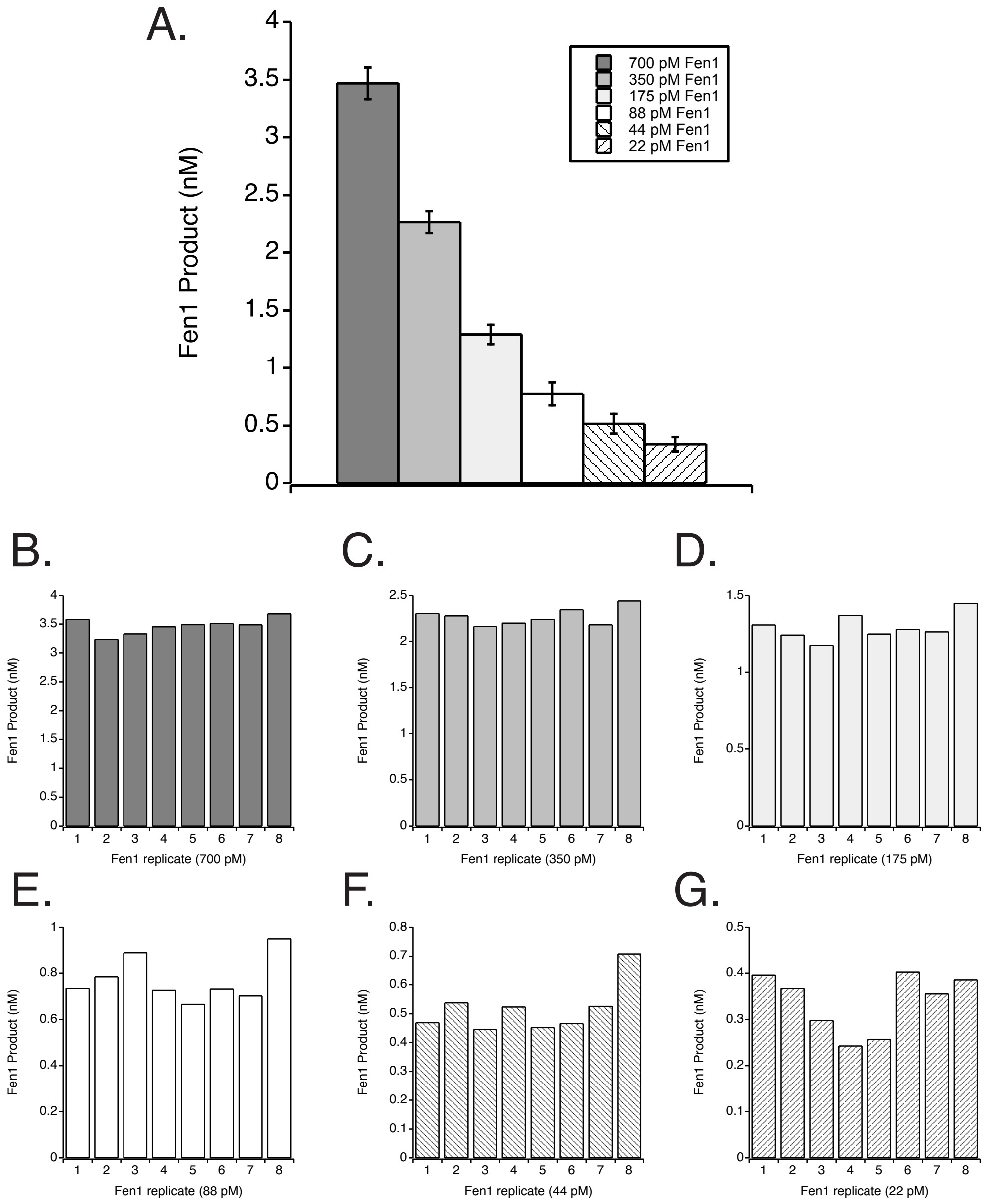

Supplemental Figure 2. Reproducibility of Fen1 cleavage assay. The Fen1 activity assay was performed with varying concentrations of Fen1 (22 to 700 pM) and resolved by CE as described in Materials and Methods. Reactions were repeated 8 times to evaluate reproducibility. Panel A plots the average amount of Fen1 product formed (with standard deviation) for each Fen1 concentration. Data from individual replicates are shown in Panels B-G for each Fen1 concentration as follows (B) 700 pM Fen1, (C) 350 pM Fen1, (D) 175 pM Fen1, (E) 88 pM Fen1, (F) 44 pM Fen1 and (G) 22 pM Fen1.
